# Predicting *Escherichia coli* levels in manure using machine learning in weeping wall and mechanical liquid solid separation systems

**DOI:** 10.3389/frai.2022.921924

**Published:** 2023-01-04

**Authors:** B. Dharmaveer Shetty, Noha Amaly, Bart C. Weimer, Pramod Pandey

**Affiliations:** ^1^Department of Population Health and Reproduction, School of Veterinary Medicine, University of California, Davis, Davis, CA, United States; ^2^Polymeric Materials Research Department, Advanced Technology and New Materials Research Institute, City of Scientific Research and Technological Applications (SRTA-City), New Borg El-Arab City, Alexandria, Egypt

**Keywords:** dairy agroecosystems, dairy manure, liquid-solid separator, machine learning models, *E. coli* risks

## Abstract

An increased understanding of the interaction between manure management and public and environmental health has led to the development of Alternative Dairy Effluent Management Strategies (ADEMS). The efficiency of such ADEMS can be increased using mechanical solid-liquid-separator (SLS) or gravitational Weeping-Wall (WW) solid separation systems. In this research, using pilot study data from 96 samples, the chemical, physical, biological, seasonal, and structural parameters between SLS and WW of ADEM systems were compared. Parameters including sodium, potassium, total salts, volatile solids, pH, and *E. coli* levels were significantly different between the SLS and WW of ADEMS. The separated solid fraction of the dairy effluents had the lowest *E. coli* levels, which could have beneficial downstream implications in terms of microbial pollution control. To predict effluent quality and microbial pollution risk, we used *Escherichia coli* as the indicator organism, and a versatile machine learning, ensemble, stacked, super-learner model called E-C-MAN (*Escherichia coli–*Manure) was developed. Using pilot data, the E-C-MAN model was trained, and the trained model was validated with the test dataset. These results demonstrate that the heuristic E-C-MAN ensemble model can provide a pilot framework toward predicting *Escherichia coli* levels in manure treated by SLS or WW systems.

## Introduction

In confined agroecosystems such as intensive dairy farms, significant quantities of animal wastes are generated that need to be managed efficiently (Van Horn et al., 1994; Meyer et al., 1997; Neufeld et al., 2017). For example, about half a million cattle can produce in excess of 27,000 tons of animal waste per day within an area of < 26,000 square kilometers (Popova and Morra, 2017). On one hand, these large quantities of animal waste and farm effluents are a good source of nutrients, effluents also poses risk to water and environment. With an increased interest in the application of treated manure to grow forage crops as an alternative to chemical fertilizers, approaches which intend to improve recycling and uses of dairy manure, bring benefits in terms of economic and environmental perspectives (Neufeld et al., 2017; Popova and Morra, 2017). Even though multiple challenges exist toward handling large quantities of dairy effluents as they could negatively contribute to water contamination, air pollution, greenhouse gas emission, and the spread of antimicrobial resistant and pathogenic bacteria, which could have human, animal, and One Health implications (Owen and Silver, 2015; Sharma et al., 2017; Vadas et al., 2017; Niles and Wiltshire, 2019), there are multiple opportunities and benefits of using manure as fertilizers.

The interaction of manure management practices with environmental, human, and animal health, has gained increased attention, and such practices must be constantly assessed to develop research and educational programs that implement alternative effluent management techniques (Meyer et al., 1997). These techniques include manure digestors, settling ponds, evaporation ponds, and solid-liquid separators (Meyer et al., 2004; Liu et al., 2017). Most of these techniques, often implemented in combination, aim to reduce greenhouse gas emissions, eutrophication stress, odor, nutrients and microbial communities; and provide options to recycle dairy effluents for beneficial purposes (Zhang and Westerman, 1997; Wang et al., 2020).

The separation of solids manure from flush manure effluents in a dairy farm can increase the efficiency of the manure management process by reducing the excessive total solids, volatile solids, and microbial communities in flushed manure. This can assist in reducing the loads in downstream lagoons and greenhouse gas emission, and improve pumping efficiency, manure uses for irrigation purposes (Mukhtar et al., 2011; Neuhaus, 2020; Ellison and Horwath, 2021). A solid-liquid separation system produces two streams: (1) solid manure stream, which is composted and dried, and used for fertilizing crops and bedding material for dairy farms; (2) liquid manure stream often used for irrigating cropland with manure enriched with nitrogen and phosphorus (Mukhtar et al., 2011; Vanotti et al., 2020; Wu and Zhong, 2020). Common approaches for separating the manure solids from the liquid stream include sedimentation or gravitational settling and mechanical screening of solids (Mukhtar et al., 2011).

**Figure 1:**
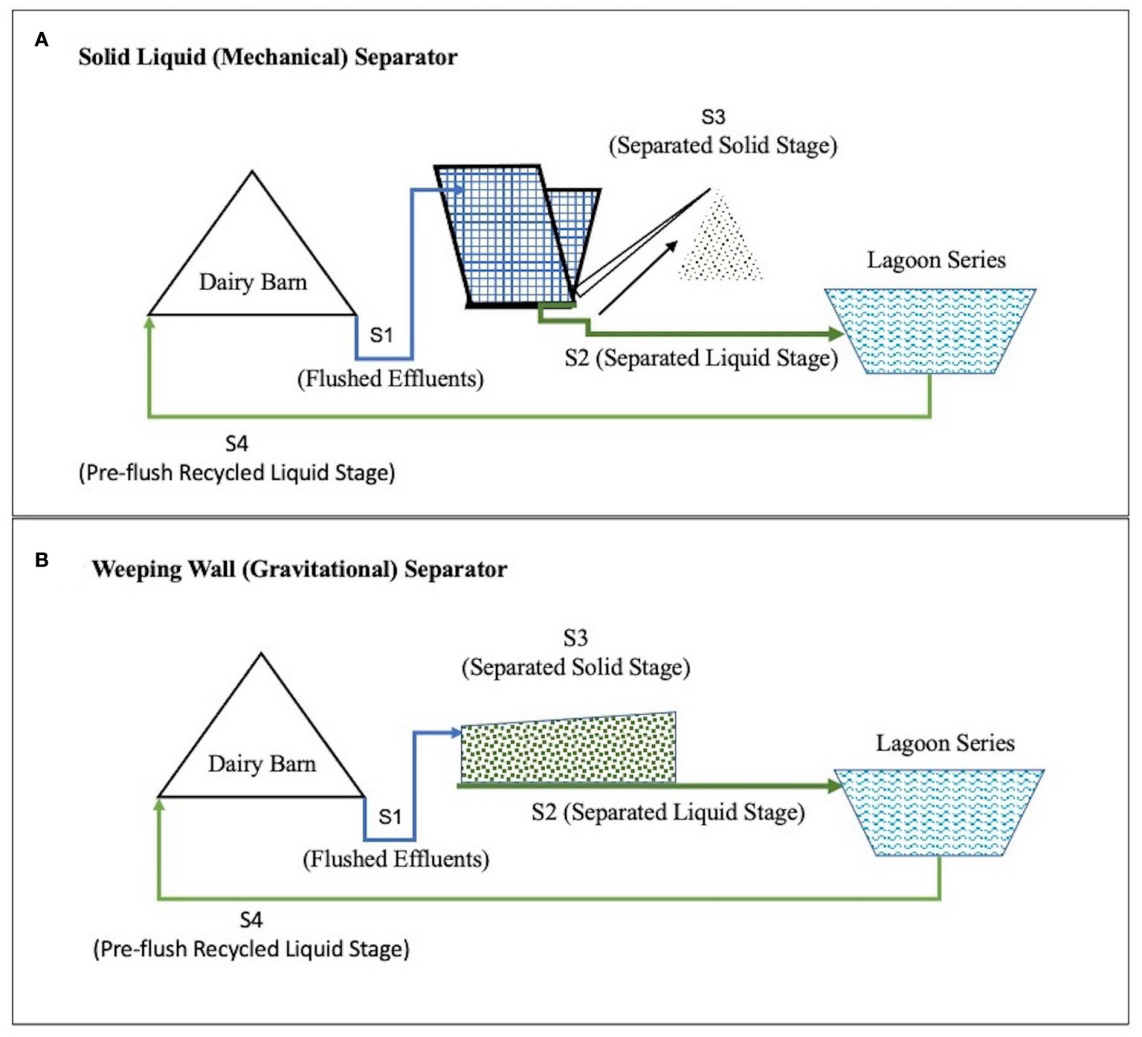
Conceptual flow of manure in the two comparative Dairy Effluent Management Systems found on two partner farms from Central California, USA. The Dairy Effluent Management System in part **(A)** includes the Mechanical Solid Liquid Separator (SLS) System and the Effluent Management System in part **(B)** includes the gravitational Weeping Wall (WW) System.

A mechanical solid-liquid separator with inclined screens and conveyor scraper separators is called the SLS (Solid Liquid Separator) System. A gravity separation system with a settling basin and a large dewatering surface area is called a WW (Weeping Wall) System. These are two commonly used solid separation techniques in the dairy farms of California, USA (Meyer et al., 2004; Mukhtar et al., 2011). In both the SLS and the WW effluent management systems, the dairy waste goes through multiple stages (Figure 1).

These stages include Stage 1 (S1), Flushed Effluent Stage (effluents are collected after flushing the barns and prior to separating the solid fraction), and Stage 2 (S2), Separated Liquid Stage, where liquid fraction is separated from solid fraction) and liquid fraction is stored in lagoons. In Stage 3 (S3), Separated Solid Stage, solid fraction of manure separated from liquid and solid is dried and composted, and Stage 4 (S4), where liquid manure (after solid settling in lagoons are recycled to dairy manure to flush dairy barn manure.

Previous studies have demonstrated that the SLS can reduce the Total Solid (TS) and Volatile Solid (VS) components from the effluent slurry by 60.9 and 62.8%, respectively (Chastain et al., 2001). Further, in combination with a two-chambered settling basin and a lagoon arranged in series, the SLS System was shown to reduce the TS by up to 93% and the VS by up to 95.6% from the dairy effluents (Chastain et al., 2001). In a single stage WW System, results shown to reduce TS by 49–63%, (Meyer et al., 2004) whereas a two-stage WW System reduced TS by 35% and VS by 40% (Mukhtar et al., 2011). The mean electrical conductivity, potassium, calcium, sodium, and chlorine soluble nutrient concentrations in a single stage WW remained unchanged between the incoming and outgoing stages of the WW System (Meyer et al., 2004).

Compared to the physical and chemical characteristics, there is limited research investigating the effect of the SLS and WW Systems toward reducing the risk of pathogenic bacteria in the dairy agroecosystems, though other forms of solid separation systems have shown variable but occasionally promising results (Boutilier et al., 2009; Liu et al., 2017; Wang et al., 2019). For example, one study demonstrated that dairy manure management practices that separate solid from liquid waste effectively reduced *E. coli* concentrations (Howard et al., 2017). Another non-comparative study on the WW ADEM demonstrated that it reduced *Cryptosporidium* oocysts, and the authors explained that this could be due to the separation of the oocysts in the liquid component of the manure (Hutchison et al., 2005). Such studies in related systems provided clues and directions for our study and its potential results.

In spite of the limited comparative studies in these specific ADEMS, it is well documented that implementing treatment methods, effective in reducing potentially pathogenic bacteria in manure, is important because many outbreaks of gastroenteritis across humans and animals have been related to livestock operations (Liu et al., 2017). Fecal coliforms including *E. coli* are common indicator organisms that are used to assess water quality and determine the presence of pathogenic microorganisms that may cause illness and disease in exposed human and animal populations (Boutilier et al., 2009). Previous studies showed that animal waste is one of the major sources of indicator organisms/E. coli in environment (Malakoff, 2002; Pandey and Soupir, 2012a). Observing the concentrations/loads of fecal coliforms, and E. coli (indicator organisms) in ambient water is often used to determine the microbial pollution in recreational and drinking water, and these strategies helps in protecting public health, and identifying the water bodies with potential pollution. Various government agencies including U.S. Environmental Protection Agencies (U.S. EPA) monitor *E. coli* levels in rivers, and lakes to determine the microbial water quality (Pandey et al., 2012b; Pandey and Soupir, 2013). Though there is much debate regarding the ability of indicator organisms to determine the human health risks caused by pathogenic bacteria, indicator organisms are widely used to determine microbial quality of water, and food. In environment, it is often challenging to determine the source of pathogens (animal waste, wildlife excreta, animal waste), animal waste is considered to be a leading source of microbial pollution in environment including ambient water (Malakoff, 2002; Dickerson et al., 2007; Pandey et al., 2014). In addition to indicator organisms, microbial source tracking to find the origin of fecal coliform are also used to reduce to public health risks (Scott et al., 2002; Grave et al., 2007; Ibekwe et al., 2011; Ma et al., 2014).

To advance our existing understanding in manure management, in this study, we attempted to bridge the prevailing knowledge gaps regarding the SLS and WW Systems. In this pilot scale study, we attempted to: (1) compare the similarities and differences in chemical, physical, and biological parameters across Dairy Effluent Management Systems containing the two solid separation systems (SLS and WW); and (2) create a versatile machine learning model using these parameters to predict the *E. coli* risk across the SLS and WW Systems in the dairy effluent systems from two farms in California, USA.

## Materials and methods

### Sample collection

In this pilot-scale study, manure characteristic data were collected from two representative dairy effluent management systems from two partner dairy farms in Central California, USA. While one of the effluent management systems included the SLS solid separation system, the other encompassed the gravitational WW solid separation system. Within each effluent management system in the two farms, samples were collected from the four stages, S1–S4 that have been described in the introduction (Figure 1). Each of the samples were collected from the top layer of the various stages and placed in independent tubes. Subsequently, the samples were maintained on ice during transportation, and stored in a cold room (4°C) upon arrival at the laboratory (Li et al., 2014), till they were processed for various parameters. Accordingly, a total of 96 manure samples were collected using a balanced sampling design for this pilot study. Forty-eight samples were collected from the Dairy Effluent Management System containing the mechanical SLS system, while another 48 samples were collected from the Management System containing the gravitational WW system. Within each of these two systems, a total of 24 samples were collected from each of the four stages, S1–S4. These samples were collected across two seasons. A total of 32 samples were collected in Spring, between March and May of 2019, whereas 64 samples were collected in Summer, during the month of June 2019.

### Chemical, physical and biological parameters

A set of chemical, physical, and biological parameters were calculated for each of the collected samples.

#### Escherichia coli

Liquid samples (from Stages S1, S2, and S4) were collected and homogenized. Subsequently, 1 ml of each sample was aliquoted and diluted with 9 ml of Millipore-filtered water. On the other hand, the solid samples (from Stage S3) were prepared by diluting 5 grams of the solid effluent in 10 ml of Millipore-filtered water. The diluted samples were homogenized by vortexing for 2 min. These samples were serially diluted ( × 10, × 10^−1^, 10^−2^× 10^−3^, × 10^−4^) using Millipore-filtered water, and 1 ml of the serially diluted samples were processed through a membrane filtration technique following the EPA method 1,603 (EPA, 2002). A VP-300 Vacuum Pump was used to create vacuum and a flow rate of 10 ml/cm^2^ /min. An MCE White Membrane/Black Grid Membrane with 0.22 μm pore size and 28 cm^2^ area (Sigma-Aldrich, St. Louis, Missouri, USA) was used for filtering the study samples, and subsequently, the filter paper was placed in a prepared modified mTEC Agar (Difco, Sparks, MD, USA) media in a Petri dish. The Petri dish with filter paper was placed in an incubator at 37°C for 24 h. Since this medium is selective, it can distinguish *E. coli* from other microorganisms. The presence of thermotolerant *Escherichia coli* was demonstrated by a chromogenic reaction, i.e., pink colonies, which were enumerated as Colony Forming Units per milliliter (CFU/ml). All the samples were filtered after fresh dilution. Each sample was analyzed in duplicates, and the experiment was repeated three times.

#### Total solids and volatile solids

Total Solids (TS) and Volatile Solids (VS) were measured using the Ignition Method (Clesceri et al., 1998). Initially, the empty dry crucible weight (W_1_) was measured, and subsequently, an ~10 ml or 10 g sample was placed in the crucible. Then, the combined weight of the wet sample and crucible was measured (W_2_). The sample was heated at 104°C for 16 h, and then, the weight of dried residue and crucible was measured (W_3_). Subsequently, the TS were measured using the Eq. (1). For measuring VS, the samples that had been dried at 104°C were placed in a furnace for 4 h at 500°C. The combined sample and crucible weight was measured after ignition (W_4_). Subsequently, the VS were measured using Eq. (2).


(1) $$\begin{array}{l} TS=\;\frac{W_{3}-W_{1}}{W_{2}-W_{1}} \end{array}$$

(2) $$\begin{array}{l} VS\;=\;\frac{W_{3}-W_{4}}{W_{2}-W_{1}} \end{array}$$

#### Chemical and physical characteristics

The nitrate, chloride, potassium, calcium, sodium, and total salt content, along with electrical conductivity and pH were measured for each sample. For liquid samples (collected from Stages S1, S2, and S4), 5 ml of each sample was transferred to an Eppendorf tube and centrifuged at 5,000 rpm (~2,432 g)for 5 min to separate any suspended solid content. For solid samples (collected from Stage S3), 1 gm of each sample was homogenously dispersed in 5 ml of water for 1 h and then separated by centrifugation at 5,000 rpm (~2,432 g) for 5 min. Subsequently, 500 μL of supernatant was taken and placed on sensors for measuring ions. Sensors for measuring nitrate, potassium, calcium, and salt ions, as well as electrical conductivity and pH, were obtained from Horiba (Horiba Limited, Japan). Each sensor was calibrated prior to measurement using known standards of nitrate, potassium, calcium, total salts, conductivity solution, and pH. All the measurements were conducted at room temperature and repeated three times for each sample.

### Comparing dairy effluent characteristics across systems, stages, and seasons

Analysis of Variance (ANOVA) tests were used to determine statistically significant differences amongst the chemical, physical, and biological parameters between the various systems, stages, and seasons for the continuous variables. Using the “arsenal” package in “R” statistical software version 4.0.3, a parameter was considered statistically significant if the *p*-value was≤ 0.05. Subsequently, in order to visualize the correlations between the various chemical, physical, and biological parameters, a correlogram (i.e., correlation matrix) was constructed using the “corrplot” package in the “R” statistical software version 4.0.3 (Wei et al., 2017). The visualization in the correlogram was conducted by employing a color-coded heat map matrix.

### Machine learning models

By using the various chemical, physical, biological, structural, and seasonal parameters, an assortment of machine learning models were built and compared to predict *E.coli* levels in dairy effluents using various packages through the “Caret” library in “R” (Kuhn, 2008). The dataset was (a) preprocessed to make it machine readable, (b) split into training and test datasets, and subsequently, (c) used to build and compare various machine learning base-learner and super-learner algorithms.

#### Creating a machine-readable dataset

Before building the models, the dataset was described and processed to make it machine-readable. Initially, the relationship between each of the chemical, physical, and biological parameters with the outcome variable, i.e., *E. coli* levels (CFU/ml), were visualized. The continuous predictor variables were plotted using smoothened scatter plots, whereas the categorical predictor variables were plotted using box-and-whisker plots. Subsequently, the dataset was analyzed for zero and near-zero variance predictors, linear dependencies amongst predictors, and highly correlated predictors. Predictors that have a single unique value, i.e., zero variance predictors, or predictors that have a handful of unique values that occur with very low frequencies, i.e., near-zero variance predictors, could cause some machine learning models to crash or create an undue bias. Linear dependencies between predictor variables should be identified to remove redundant variables from the dataset.

Highly correlated predictors could either improve or decrease the performance of select machine learning algorithms, and thus, it is important to identify such predictors prior to building comparative models. In addition to individually scanning for zero variance predictors, linear dependencies, and highly correlated predictors, the model variables were also subject to select normalizing transformations using the BestNormalize R package in order to improve the efficiency of the regression machine learning algorithms (Peterson and Peterson, 2020). After transformation, the success of the normalizing transformation was determined by various mechanisms, including creating visual plots, using the Shapiro test, and by calculating the skewness value.

#### Splitting the dataset into training and test datasets

The machine-readable dataset was split into a training and test dataset. The probability-based *createDataPartition* function in the R package *caret* (Kuhn, 2008) was used to obtain a 80:20 balanced split of the dataset. Using the training dataset, a Missing Data model, a Dummy Variable model, and a Transformation model were developed. While the Missing Data model, which used the K-nearest neighbor (knn) method, was used to impute missing predictor values and simultaneously, center and scale all the predictor values. A Dummy Variable model was developed to create one-hot coded variables for the categorical predictors, and a Transformation model was used to transform all the predictor values into a range from 0 to 1. Subsequently, using these three models, missing data was imputed, dummy variables were created for categorical predictors, and predictor values were transformed for both the training dataset and the test dataset, respectively.

#### Machine learning algorithms

More than 30 machine learning models, including 28 base-learner and independent regression models from seven different families of algorithms (Supplementary Table 1), and multiple super-learner ensemble stacked models were constructed and compared. The 28 independent models were included from the following families: generalized linear models, random forest models, boosting models, support vector machine (SVM) models, multivariate adaptive regression splines (MARS), neural networks, and partial least square models. Each of these models were trained with the training dataset. In order to optimize each of these models, the individual models were cross-validated, and the constituent model hyper parameters tuned by using a repeated k-fold cross validation (repeated CV) method with 10 folds and 20 repeats.

#### Super-learner ensemble models

Multiple stacked super-learner ensemble models were also constructed by using combinations of the base-learner regression models in multiple frameworks, such as a Generalized Linear Model (GLM) framework. The GLM ensemble were built using the Root Mean Square Error (RMSE) metric, and cross-validation was performed by tuning the parameters using a repeated k-fold cross validated method with 10 folds and 20 repeats. Amongst multiple combinations, the ensemble trials included GLM ensembles of all the 28 base-learner models, random forest (RF) ensembles of all the 28 base-learner models, subsets of the base-learner models, and the seven best-fit models from the seven different families of machine learning models used in the present study.

#### Choosing the model with the highest predictive capability

The Root Mean Square Error (RMSE) metric, the most commonly used method for evaluating and comparing a models predictive capabilities (Kuhn and Johnson, 2013), was used to select the relatively best model. This metric is a function of the model residuals, and the square root of the Mean Square Error (Kuhn and Johnson, 2013). The predictive capability of a model is negatively correlated with its RMSE value, i.e., the lower the RMSE values, the higher the predictive capability of the model. For the final model that was chosen, the values were reversed transformed.

#### Relative importance of the individual predictors in the machine learning model

Subsequently, the relative importance of individual predictors was calculated for the best fit independent base-learner model using the LOESS (Locally Estimating Scatter Plot Smoothening) R-squared variable importance method, where the R^2^ statistic is calculated against the intercept only null model. In order to evaluate the analytical authenticity of the results, the top two predictors were removed from the data, and the model was rerun.

## Results and discussion

### Chemical, physical, and biological parameters

Chemical, physical and biological parameters were calculated for each of these 96 samples (Table 1). The Analysis of Variance (ANOVA) tests demonstrated that potassium, pH, *E. coli*, sodium, total salts, and volatile solid levels, in descending order of significance, were statistically different (*p*≤0.05) between the Management Systems containing the SLS and the WW Systems. Calcium, nitrates, sodium, potassium, total solids, volatile solids, and *E. coli* levels were significantly different in at least one of the four different stages, S1, S2, S3, or S4. Similarly, calcium, nitrates, sodium, potassium, total solids, volatile solids, *E. coli*, and total salt levels were also significantly different in at least one of the eight different combinations of systems (*n* = 2) and stages (*n* = 4). Only *E. coli*, sodium, potassium, and volatile solid levels were independently and significantly different between at least one of the two systems, one of the four stages, as well as one of the eight system-stage combinations. None of the variables were significantly different during either the Spring or the Summer season.

**Table 1:** Descriptive effluent characteristics in dairy effluent management systems.

**Parameter**	**Dairy effluent management systems**							
	**Mechanical solid-liquid separator (SLS)**				**Gravitational weeping wall solid separator (WW)**			
	**Stages**				**Stages**			
	**S1**	**S2**	**S3**	**S4**	**S1**	**S2**	**S3**	**S4**
Total salts^*#^ (%)	0.12 (0.11)	0.11 (0.11)	0.04 (0.04)	0.12 (0.09)	0.06 (0.03)	0.08 (0.06)	0.07 (0.08)	0.05 (0.01)
Calcium^#^ (mg/mL)	488.31 (132.84)	524.44 (131.67)	196.50 (140.58)	505.56 (104.69)	378.89 (70.73)	418.50 (68.92)	293.89 (79.75)	413.61 (115.61)
Nitrate^#^ (mg/mL)	667.50 (263.35)	574.17 (191.16)	208.17 (263.6)	562.78 (196.32)	594.44 (241.93)	565.83 (223.81)	397.22 (179.52)	702.78 (332.06)
Sodium^*#^ (mg/mL)	481.11 (171.60)	549.67 (269.43)	201.08 (181.83)	536.11 (275.50)	370.83 (99.65)	383.06 (77.78)	262.33 (81.57)	319.72 (78.296)
Potassium^*#^ (mg/mL)	805.28 (228.25)	786.03 (171.54)	218.42 (95.85)	683.89 (219.60)	1,412.50 (512.82)	1,359.17 (471.60)	835.28 (255.43)	1,439.72 (503.24)
pH*	7.10 (0.18)	7.11 (0.16)	7.06 (0.11)	7.16 (0.10)	7.17 (0.27)	7.24 (0.14)	7.24 (0.27)	7.30 (0.34)
EC (mS/cm)	1.28 (0.13)	1.32 (0.14)	1.23 (0.13)	1.28 (0.16)	1.27 (0.11)	1.34 (0.08)	1.34 (0.08)	1.26 (0.07)
TS^#^ (%)	2.98 (3.41)	4.87 (6.44)	32.17 (29.77)	2.83 (3.24)	1.40 (0.63)	2.44 (4.29)	21.31 (13.72)	4.92 (6.82)
VS^*#^ (%)	1.50 (2.83)	2.39 (5.73)	16.77 (9.97)	1.45 (2.56)	0.56 (0.36)	0.50 (0.40)	6.04 (4.33)	1.80 (2.95)
*E*.coli^*#^ (CFU/mL)	1,727.99 (1,197.33)	1,691.25 (1,065.55)	150 (88.99)	483.06 (524.22)	3,775 (2,768.7)	2,077.36 (1,684.84)	112.33 (70.02)	2,491.39 (2,286.2)

The values are described as mean values (and standard deviation in brackets). An asterix (*) superscript indicates that the parameter is statistically significantly different between samples from SLS and WWS, whereas a hashtag (#) superscript indicates that the parameter is significantly different in at least one of the eight combined system (SLS vs. WW) and stage (S1, S2, S3, or S4) combinations. A parameter is considered to be significantly different if *p*≤*0.05*.

CFU/ml, Colony Forming Units per milliliter; TS, Total Solids; VS, Volatile Solids; EC, Electrical conductivity; mS/cm, millisiemens per centimeter.

### Descriptive correlogram

**Figure 2:**
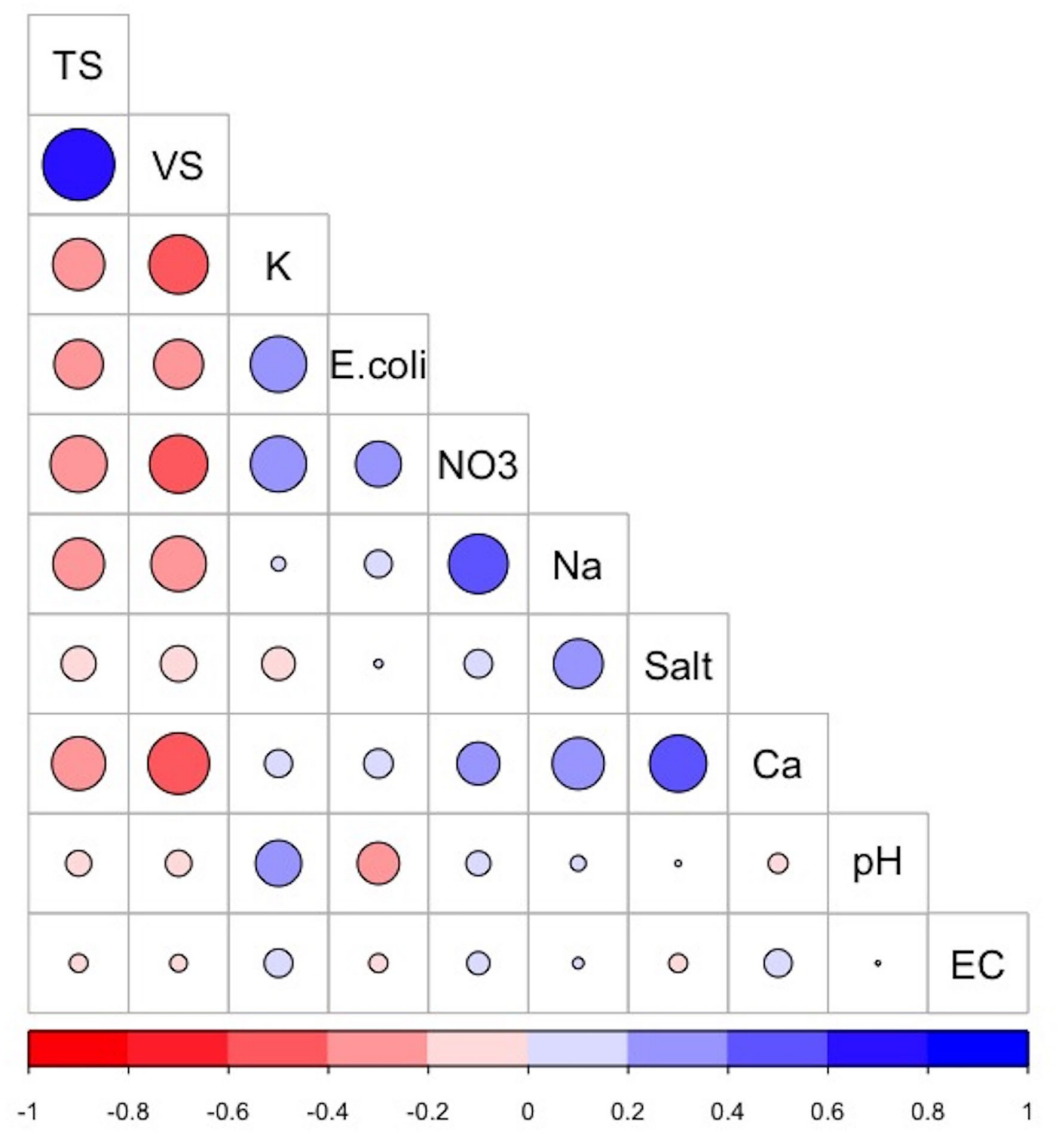
Correlogram showing the correlation matrix between the various dairy effluent parameters. The positive correlations and negative correlations are colored **blue** and **red**, respectively. The intensity and size of the circle are proportional to the correlation coefficients. Abbreviations used: TS, Total Solids; VS, Volatile Solids; K, Potassium; NO3, nitrates; Na, Sodium; Ca, Calcium; Salt, Total Salts; EC, Electrical Conductivity. The image was generated using the “Corrplot” package in R version 4.0.3.

The correlation matrix (Figure 2) visualizes the strength and direction of correlation between the various chemical, physical, and biological parameters analyzed from the collected dairy effluent samples. The *E. coli* levels were positively correlated with potassium (+0.39) and nitrates (+0.26), and negatively correlated with total solids (−0.30) volatile solids (−0.30), and pH (−0.22). The sodium levels were positively correlated with nitrates (+0.44), calcium (+0.33), and total salts (+0.3), and negatively correlated with total solids (−0.3) and volatile solids (−0.3). The total solids were positively correlated with volatile solids (+0.64), and negatively correlated with nitrates (−0.4), calcium (−0.36), potassium (−0.33), sodium (−0.33), and *E. coli* levels (−0.30). The volatile solids were positively correlated with total solids (+0.64), and negatively correlated with calcium (−0.47), potassium (−0.43), nitrates (−0.42), sodium (−0.38), and *E. coli* (−0.3). Other variables which displayed positive correlations are nitrates and sodium (+0.44), total salts and calcium (+0.4), and nitrates and potassium (+0.39).

### Machine learning models

#### Creating a machine-readable dataset

**Figure 3:**
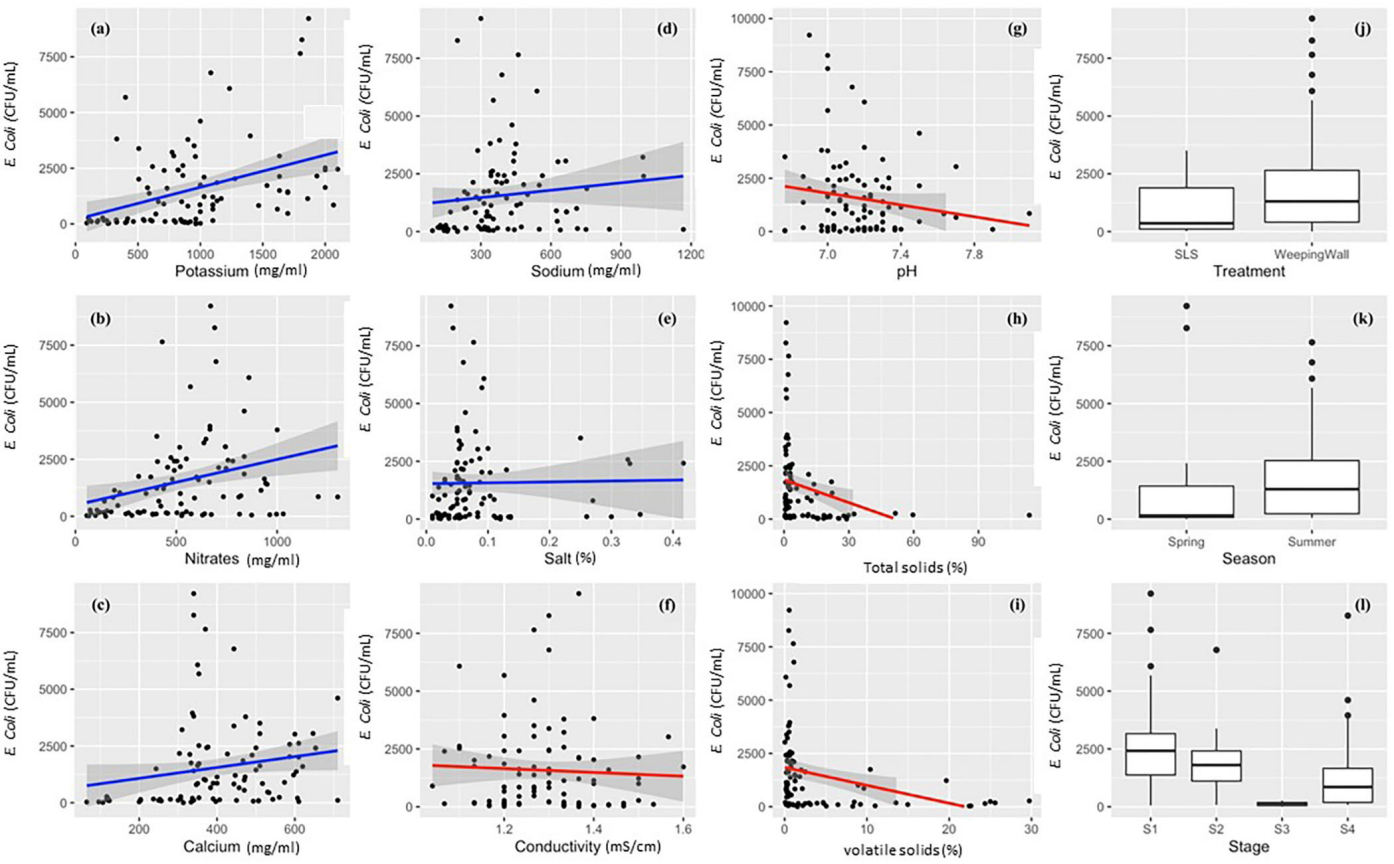
Descriptive univariate graphs of model variables visualizing the relationship between the individual predictor variables and the outcome variable (live *E. coli* levels). The continuous variables have been mapped as scatterplots in graphs **(a–i)**, which include **(a)** potassium, **(b)** nitrates, **(c)** calcium, **(d)** sodium, **(e)** total salts/salt, **(f)** electrical conductivity, **(g)** pH, **(h)** total solids/TS, and **(i)** volatile solids/VS. Each univariate scatterplot has been fit using a smoothened conditional mean in the form of Generalized Linear Models (GLM) with a 95% confidence interval. A blue line indicates a positive relationship **(a–e)**, red line indicates a negative relationship **(f–i)**, and the gray shaded area denotes the 95% confidence interval for each GLM that has been fit to the data points in the graph.

Machine learning models were built to predict *E. coli* levels across dairy effluent management systems that included either the SLS or WW solid separation systems. To develop a better understanding of the nature of variables that was going to be used in the final model, the univariate relationship between individual predictor variables and the l *E. coli* levels were visualized using scatter plots fitted with Generalized Linear Models (with 95% confidence interval) for continuous variables, and Box-and-whisker plots for categorical variables (Figure 3). Whereas, potassium, nitrates, calcium, and sodium demonstrated a positive linear relationship with *E. coli* levels; total solids, pH, electrical conductivity, and volatile solids demonstrated a negative linear relationship. The Box-and-Whisker plots demonstrated that the management systems with the gravitational WW had relatively higher *E. coli* levels compared to the management systems with the mechanical SLS (Figure 3j), which was reconfirmed to be significantly higher in the ANOVA test (Table 1). It should be noted here that this result was at the level of the management regime (WW vs. SLS) and not at the finer stage setting. With respect to seasons, *E. coli* levels were higher in the summer relative to the spring season (Figure 3k), but this difference was found to be statistically insignificant in an ANOVA test. Stage S3 had the lowest levels of *E. coli* with a highly compact distribution spread (Figure 3l).

Subsequent analysis demonstrated that the dataset did not have any zero or near-zero variance predictors. In addition, there were no linear dependencies or variable combinations in the dataset, and none of the model variables were highly correlated, i.e., correlated more than 0.75. In order to increase the efficiency of the downstream analysis, especially parametric models, normalizing transformations were performed on the model variables. Whereas, *E. coli*, total salts, potassium, and total solids were transformed using the “OrderNorm” normalizing transformation (Bartlett, 1947), an ordered quantile normalizing transformation; Volatile Solids was transformed using the Standardized Box Cox normalizing transformation (Box and Cox, 1964); sodium was transformed using the ArcSinh normalizing transformation, and pH was transformed using the Standardized Yeo-Johnson normalizing transformation (Yeo and Johnson, 2000).

#### Splitting the dataset into training and test datasets

The dataset was split into a training and test datasets. In the training dataset, missing data was imputed for a total of eight data points each amongst the total solids and volatile solids variables. There was no missing data in the test dataset. Two, four, and two dummy variables were created for systems, stages, and seasons, in both the training and the test datasets. Finally, the data was transformed by centering, scaling, and restricting the predictor values to a range between 0 and 1.

#### Machine learning algorithms

**Figure 4:**
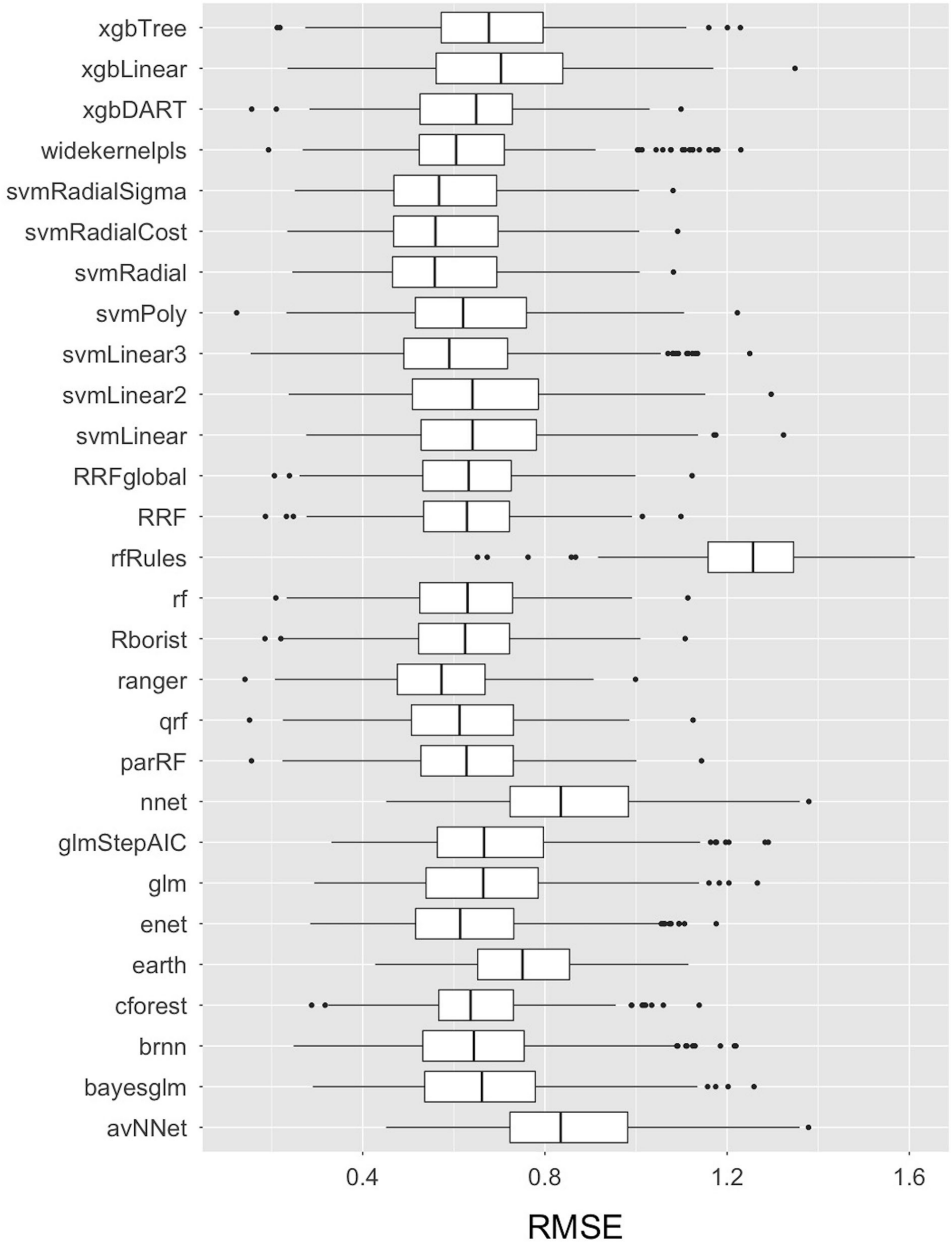
Box-and-Whisker plots comparing the Root Mean Square Error (RMSE) values of the 28 base-learner independent machine learning algorithms used to train the data in predicting live *E. coli* levels in Dairy Effluent Management Systems. The plots display the median with a horizontal line, the 25th and 75th percentiles with the lower and upper box limits, the extreme observations with the bars, and the outlier data as dots. These RMSE values pertain to transformed data.

More than thirty Machine Learning algorithms, including 28 base-learner regression models from seven different families of algorithms, and more than two super-learner stacked GLM (Generalized Linear Model) and RF (Random Forest) ensemble models were built and compared using the RMSE accuracy measure for the transformed *E.coli* variable in the training dataset. The code is available on Github and will be made available upon request. The comparative RMSE measures for the 28 base-learner independent regression models are plotted as Box-and-whisker plots in Figure 4. Amongst the 28 independent regression machine learning models, the Ranger model (with model parameters: mtry = 15, split rule = extra trees, and minimum node size = 5) had the lowest mean RMSE value of 0.57, followed by the SVM Radial model (with model parameters: epsilon = 0.1 and cost = 2, and hyperparameters: sigma = 0.04) with a mean RMSE value of 0.58. In addition to these two machine learning models, other base-learner models with a relatively low RMSE value included the SVM Radial Sigma (RMSE = 0.58), SVM Radial Cost (RMSE = 0.59), and the qrf (RMSE = 0.61) models. The results of the base-learner models demonstrated that the Random Forest and Support Vector Machine (SVM) family of machine learning algorithms performed the best amongst the seven families for our study dataset.

When the variable importance was calculated by using the LOESS smoother non-parametric model, stage S3 and total solids were found to be the two most important predictor variables (Table 2). Amongst the various predictors, Stage S3, i.e., Separated Solid Stage (i.e., solid fraction of the separated effluents) had the highest predictive value. Statistically, this was likely because it was relatively lower in both its central tendency and dispersion measures, as well as offering minimal overlap with the other stages (Figure 3l). The relatively lower *E. coli* levels in S3 is hypothesized due to the generation of heat during the separation process (Howard et al., 2017) and/or the separation of *E. coli* in the separated liquid fraction (S2) of the manure. These hypotheses will need to be tested in future studies. The second most important predictor, total solid, is likely related to the Stage S3 variable, since the TS values in Stage S3 is high, which means that the moisture content was low. Due to this relationship between S3 and TS, the hypotheses that need to be tested in further studies are also similar, including the generation of heat during the process, which leads to high total solids value. Statistically, the relationship between total solids and *E. coli* follows a negative binomial distribution, and thus, there is high variability of *E. coli* levels for lower total solid content, but low variability and levels of *E. coli* for higher total solids content. When the same best fit independent base-learner model, i.e., Ranger, was rerun without these two predictor variables, the performance dropped by more than 15% (from an RMSE value of 0.57 to 0.66); thus, demonstrating the importance of these top two predictors.

**Table 2:** Variable importance of the regression predictors, scaled to a maximum value of 100, calculated by fitting a non-parametric LOESS R-squared model between the outcome and the prediction variables.

**Variable**	**Importance**
Stage: S3	100
Total solids	86.25
Nitrates	79.89
Volatile solids	72.75
Calcium	70.39
Sodium	70.04
Potassium	46.43
Total salts	45.09
Season: spring	44.45
Season: summer	44.45
Stage: S1	30.96
Electrical conductivity	26.79
Treatment: SLS	11.59
Treatment: weeping wall	11.59
Stage: S2	9.31
pH	5.17
Stage: S4	0.00

Values generated using the “varImp” function from the “caret” package, R statistical software version 4.0.3.

#### Super-learner ensemble models

of the multiple stacked ensemble machine learning models that were attempted, a Generalized Linear Model comprising of 26 base-learner algorithms (Supplementary Table 1) from seven different families, including three Generalized Linear Models, two Partial Least Square models, eight Random Forest models, three Boosting models, six Support Vector Machine models, one Multivariate Adaptive Regression Splines model, and three Neural Network models, provided the best fit since it yielded the lowest RMSE value of 0.51 (with an R^2^ = 0.71) for the transformed *E.coli* dependent variable. The other ensemble models that were attempted, such as the 28-model GLM ensemble and the 7-model GLM ensemble, yielded an RMSE value of 0.52 (R^2^ = 0.71) and 0.56 (R^2^ = 0.67), respectively.

#### E-C-man model

**Figure 5:**
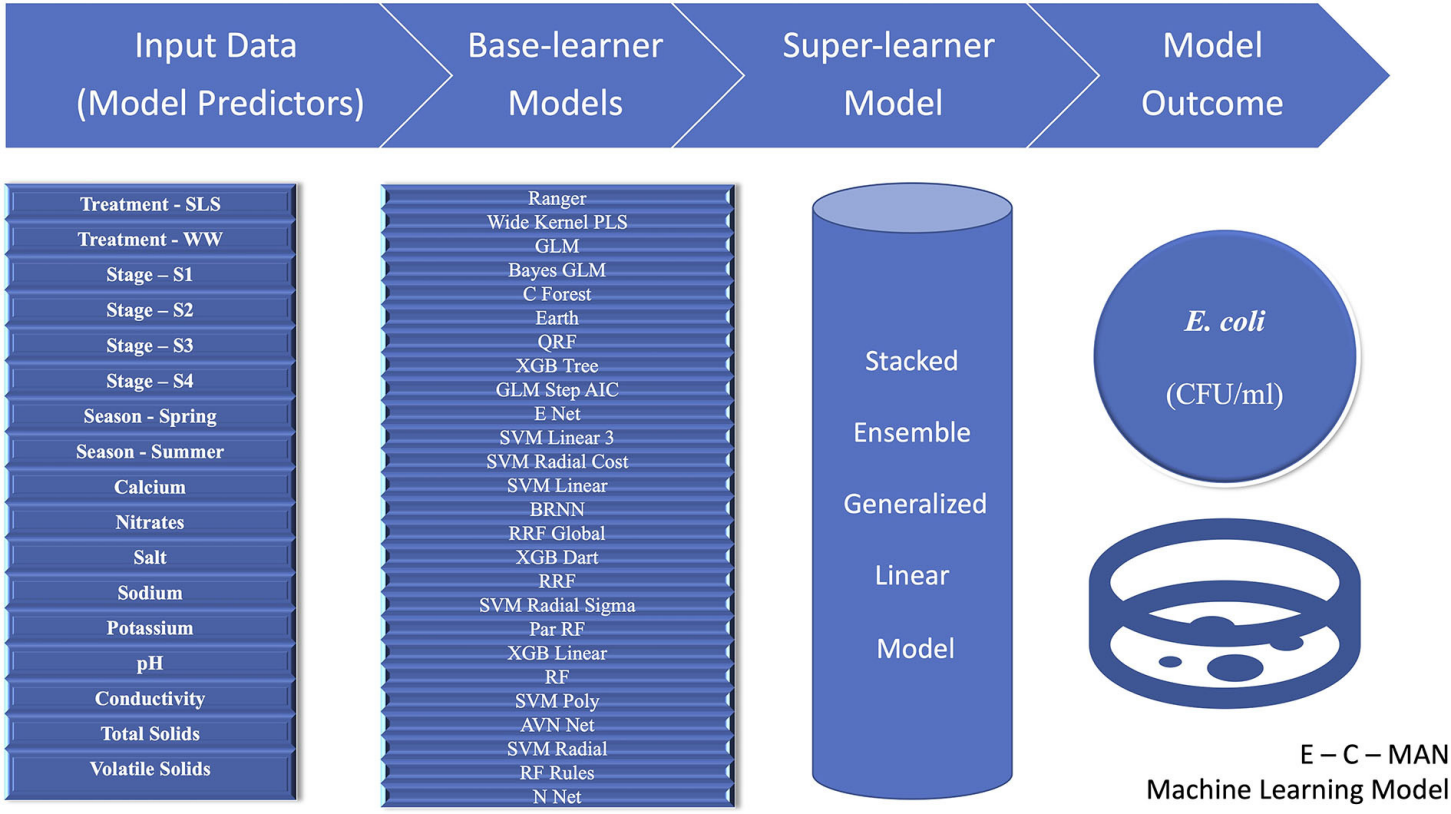
Visual representation of the E-C-MAN stacked ensemble Generalized Linear Model that was built using 17 chemical, physical, structural, and seasonal input variables to predict *Escherichia coli* levels (Colony Forming Units per milliliter, CFU/ml) in Dairy Effluent Management Systems that utilized either the gravitational Weeping Wall (WW) or the mechanical Solid-Liquid Separator (SLS) systems. The E-C-MAN model comprised of 26 base-learner regression models, whose individual outputs were stacked in a Generalized Linear Ensemble Model to predict *E. coli* levels.

Amongst all the various machine learning models that were attempted, including the various base learner regression models and the super-learner ensemble models, the GLM ensemble super-learner model with the 26 base-learner algorithms had the lowest RMSE value of 0.51 for the transformed *E.coli* variable. This was better than the 28 model GLM ensemble (RMSE = 0.52) or the best-fit independent machine learning base-learner Ranger model (parameters: mtry = 15, split rule = extra trees, and minimum node size = 5; RMSE = 0.57). Thus, we propose to name this “26 base learner ensemble” model as the “E-C-MAN” (*E. coli* Manure) Ensemble Model (Figure 5). In the E-C-MAN model, the constituent Ranger, enet, svm Radial Sigma, and cforest algorithms were found to be the most important base-learner predictors. The E-C-MAN model was further validated with the test dataset to provide an RMSE value of 0.72 (and an R^2^ of 0.65). The final values were reverse transformed to get RMSE values of 2,100 CFU/ml for the training dataset and 2,420 CFU/ml for the test dataset.

Though a previous study had built a Random Forest machine learning model to predict *E. coli* in soil amended with untreated animal manure (Pang et al., 2020), this is the first study which has used machine learning to predict *E. coli* levels directly in WW and SLS ADEMS. Using machine learning models to predict bacteria, compared to other techniques such as survival modeling, offers both advantages and disadvantages. Machine learning models are both powerful and versatile. Also, since we used a wide array of parametric and non-parametric models in our study, we were not limited by inherent assumptions associated while using a single model. In addition, machine learning models can be continuously improved with additional data. On the flip side, machine learning models are time consuming and could be error prone, especially if the input data is not accurate, reliable, or based on plausibility.

Within the scope of these advantages and disadvantages, our machine learning results demonstrate that the heuristic E-C-MAN ensemble model can be used to predict *Escherichia coli* levels in Dairy Effluent Management Systems that include the SLS or the WW solid separation systems; which can be used as an indicator to monitor effluent quality and coliform risk across the system.

### Successful completion of study objective 1: Comparing SLS and WW ADEMS using pilot data

Our study found multiple similarities and differences while using pilot data to compare the SLS and WW ADEMS in the two dairy farms of California, USA. One of the most applicable results was that the *E. coli* level in both the SLS and WW ADEMS was significantly lower in Stage S3, i.e., separated solid stage of the two ADEMS. In fact, both the ADEMS had comparable levels of *E. coli* in their S3 stage. Since the S3 stage or the separated solid component of the manure can be processed further and used for fertilization purposes, the decreased levels of *E. coli*, which was used as an indicator for measuring effluent quality and coliform risk in our study, is suggestive of lower public, veterinary, environmental, and One Health downstream challenges. However, we would like to offer a note of caution and mention that these observations need to be followed up by further studies, including studies that look at other pathogens, to ensure that the results found in this study are replicable across different pathogens and health systems.

This study also found that, when data from all the stages were merged and analyzed at the level of the management system (SLS vs. WW), the WW had significantly higher *E. coli* levels compared to the SLS. Upon taking a deeper dive into the data, we realize that this difference is likely a result of the more than the five- and two-fold higher *E. coli* levels present in Stage S4 and S1, respectively, of the WW ADEMS compared to the SLS ADEMS. For example, while WW S4 had relatively higher *E. coli* levels [mean = 2,491.39, and standard deviation = 2,286.2)], SLS S4 has lower levels (mean = 483.06, and standard deviation = 524.22). There could be multiple explanations for this difference in S4 values between the two systems. (1) it could be an inherent difference between the two systems. (2) it could be a farm specific management factor since the SLS occasionally (and rarely) dilutes their S4 with effluents from the milk plant directly, which might have ended up diluting the *E. coli* levels from the lagoons. (3) it could be a difference that randomly occurred due to chance/probability. However, in the absence of data, these and other potential causes need to be investigated further by collecting more covariant data such as the management regimes at the level of the individual farm.

At this point, it is important to note that we processed all the samples together at the end of the field season to ensure that there were minimal variations with respect to the laboratory processing of all the samples. Though this could have created a bias in downstream technical analysis, especially involving the laboratory processing for the biological (*E. coli*) parameter (Boutilier et al., 2009), we believe that the bias is likely to be uniform across the samples collected during the different systems and stages at any one chronological time of collection, and thus, not have any differential effect toward the various comparisons. In addition to the results with respect to *E. coli*, our study also found that significant differences were observed in potassium, pH, sodium, total salts, volatile solids, and *E. coli* levels between the SLS and WW systems, while the differences were found to be significant in the calcium, nitrates, sodium, potassium, total solids, volatile solids, and *E. coli* levels in at least one of the four different stages, S1–S4, across the two ADEMS.

### Successful completion of study objective 2: Building a pilot predictive machine learning model

This study also successfully created a pilot versatile machine learning model called E-C-MAN that used the chemical, physical, biological, structural, and seasonal parameters to predict the *E. coli* levels across the SLS and WW ADEMS from two dairy farms in California, USA. The model was selected after training and comparing over 30 different machine learning models, and further tested to internally validate the results. Resources permitting, we would like to build on this study by increasing our data input and externally validating the model in future studies, which will increase the prediction value of the model.

Our machine learning analysis highlighted the importance of Stage S3 (separated solids) and the total solid parameter in predicting the *E. coli* levels across the two ADEMS. Since these variables are also important for downstream applications such as fertilization, this was an important result that needs to be investigated further for public, veterinary, environmental, and One Health reasons. Though this model was a theoretical endeavor to better understand and predict the *E. coli* dynamics in SLS and WW of the ADEMS, we hope that it can also be applicable in the field with further tweaking. For example, we hope that it can be applied, either in its present or reoriented form, in systems made by the manufacturer of WW and SLS ADEMS to predict *E. coli* values using automatically calculated chemical and physical parameters, with seasonal and structural input. To conclude, we believe that this model improves the knowledge base regarding the prediction of *E. coli* as an indicator organism for effluent quality and coliform risk in alternate dairy effluent management systems, for multiple stakeholders, including farmers, researchers, scientists, funding agencies, and regulatory bodies.

## Conclusions

This study was successful in its primary goal of bridging the existing knowledge gap with respect to the mechanical Solid-Liquid-Separator (SLS) and gravitational Weeping Wall (WW) Alternate Dairy Effluent Management Systems (ADEMS), and completing both its objectives, i.e., using pilot data and (1) comparing the similarities and differences in chemical, physical, and biological parameters across the SLS and WW ADEMS, and (2) creating a versatile machine learning model using these parameters, along with structural and seasonal parameters, to predict the *E. coli* risk across the SLS and WW ADEMS from two dairy farms in California, USA. The study found that many similarities and differences exist between the SLS and WW ADEMS. In both the ADEMS, Stage S3 or the Separated Solids Stage, was found to have significantly lower *E. coli* levels, which could have important downstream implications as a manure source. With respect to the other parameters, sodium, potassium, total salts, volatile solids, pH, and *E. coli* levels were significantly different between the SLS and WW ADEMS. Further, these *E. coli* levels were used as an indicator organism to predict effluent quality and coliform risk by building an ensemble, stacked, E-C-MAN model. This study provides additional information about about the intersection between manure management and effluent quality. Given the increased pilot knowledge generated by this study on SLS and WW ADEMS, including the comparative similarities, differences, and *E. coli* prediction capabilities, we hope that future studies will build upon this pilot study to benefit the multiple stakeholders who are associated with the WW and SLS ADEMS.

## Resource identification

caret, RRID:SCR_021138.

## Data availability statement

The original contributions presented in the study are included in the article/Supplementary material, further inquiries can be directed to the corresponding author.

## Author contributions

BS: contributed to the study design, field data collection, data analytics, model generation using R, result interpretation, and drafting. NA: contribution included laboratory data generation and drafting. BW: contributed to study design. PP: contributed to study design, field data collection, and drafting. All authors contributed to the article and approved the submitted version.
