# Supplementary material for: Predicting Escherichia coli levels in manure using machine learning in weeping wall and mechanical liquid solid separation systems
Source: Front Artif Intell. 2023 Jan 4;5:921924. doi: 10.3389/frai.2022.921924 (PMC9848401; doi:10.3389/frai.2022.921924)
Supplement: Supplementary file 1 [file Data_Sheet_1.docx]

Supplementary Material

| **SUPPLEMENTARY TABLE S1: Details of 28 Machine Learning Regression Models from seven different Model Families that were used train and compare data obtained from two different types of manure management systems in order to predict *E. coli* counts within and between the systems. The constituent models of the final EC-MAN stacked model are marked with an asterix.** | | | | |
| --- | --- | --- | --- | --- |
| **Family of**  **Models** | **Model**  **Name** | **Model**  **Method** | **R Library**  **Used** | **Model Tuning Parameters** |
| Generalized Linear Models | **Bayesian Generalized Linear Model** | bayesglm* | arm | None |
|  | **Generalized Linear Model** | **glm*** | **Base R** | **None** |
|  | **Generalized Linear Model with Stepwise Feature Selection** | glmStepAIC* | MASS | None |
| Random Forests | **Random Forest** | **rf*** | **randomForest** | **mtry** |
|  | **Conditional Inference Random Forest** | cforest* |  |  |
|  | **Parallel Random Forest** | **parRF*** | **E1071, randomForest, foreach, import** | **mtry** |
|  | **Quantile Random Forest** | **qrf*** | **quantregForest** | **mtry** |
|  | **Ranger Random Forest** | **ranger*** | **E1071, ranger, dplyr** | mtry, splitrule, min.node.size |
|  | **Rborist Random Forest** | **Rborist** | **Rborist** | **predFixed, minNode** |
|  | **Random Forest Rule-Based Model** | **rfRules*** | randomForest, inTrees, plyr | mtry, maxdepth |
|  | **Regularized Random Forest** | **RRF*** | **randomForest, RRF** | **Mtry, coefReg, coefImp** |
|  | **Regularized Random Forest Global** | **RRFglobal*** | **RRF** | **Mtry, coefReg** |
| Boosting | **eXtreme Gradient Boosting** | **xgbDART*** | xgboost, plyr | nrounds, max_depth, eta, gamma, subsample, colsample_bytree, rate_drop, skip_drop, min_child_weight |
|  | **eXtreme Gradient Boosting - Linear** | **xgbLinear*** | xgboost | nrounds, lambda, alpha, eta |
|  | **eXtreme Gradient Boosting - Tree** | **xgbTree*** | xgboost, plyr | nrounds, max_depth, eta, gamma, colsample_bytree, min_child_weight, subsample |
| Support Vector Machines (SVM) | **Support Vector Machines with Radial Basis Function Kernel** | **svmRadial*** | kernlab | **sigma, C** |
|  | **Support Vector Machines with Linear Kernel** | **svmLinear*** | kernlab | tau |
|  | **Support Vector Machines with Linear Kernel** | **svmLinear2** | e1071 | cost |
|  | **L2 Regularized Support Vector Machine (dual) with Linear Kernel** | **svmLinear3*** | LiblineaR | cost, Loss |
|  | **Support Vector Machines with Polynomial Kernel** | **svmPoly*** | kernlab | degree, scale, C |
|  | **Support Vector Machines with Radial Basis Function Kernel** | **svmRadialCost*** | kernlab | **C** |
|  | **Support Vector Machines with Radial Basis Function Kernel** | **svmRadialSigma*** | kernlab | sigma, C |
| Multivariate Adaptive Regression Splines | **MARS** | **earth*** | earth | **nprune, degree** |
| Neural Networks | **Model Averaged Neural Network** | **avNNet*** | nnet | **size, decay, bag** |
|  | **Neural Network** | **nnet*** | nnet | **size, decay** |
|  | **Bayesian Regularized Neural Networks** | **brnn*** | brnn | **neurons** |
| **Partial Least Squares** | **Partial Least Squares** | **widekernelpls*** | pls | **ncomp** |
|  | **Elasticnet** | **enet*** | elasticnet | **fraction, lambda** |

**
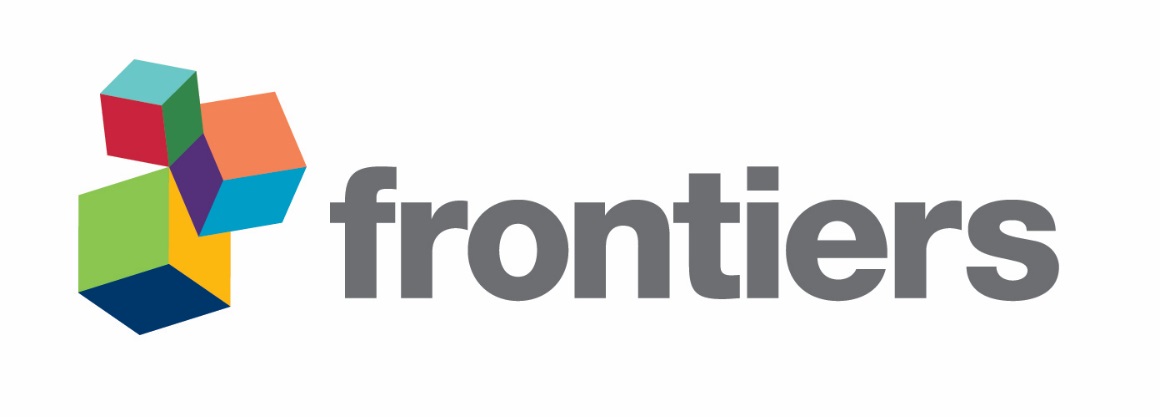
**
